# International meeting on sarcoptic mange in wildlife, June 2018, Blacksburg, Virginia, USA

**DOI:** 10.1186/s13071-018-3015-1

**Published:** 2018-08-03

**Authors:** Francisca Astorga, Scott Carver, Emily S. Almberg, Giovane R. Sousa, Kimberly Wingfield, Kevin D. Niedringhaus, Peach Van Wick, Luca Rossi, Yue Xie, Paul Cross, Samer Angelone, Christian Gortázar, Luis E. Escobar

**Affiliations:** 1 Department of Fish and Wildlife Conservation, Virginia Tech, Blacksburg, VA USA; 2 Universidad Mayor, Santiago, Chile; 3 Department of Biological Sciences, University of Tasmania, Hobart, Australia; 4 Montana Fish, Wildlife and Parks, Bozeman, MT USA; 5 Division of Immunology, Harvard Medical School, Boston, MA USA; 6 Virginia-Maryland College of Veterinary Medicine, Virginia Tech, Blacksburg, VA USA; 7 Southeastern Cooperative Wildlife Disease Study, College of Veterinary Medicine, University of Georgia, Athens, GA USA; 8 The Wildlife Center of Virginia, Waynesboro, VA USA; 9 Università degli Studi di Torino, Torino, Italy; 10 Department of Parasitology, College of Veterinary Medicine, Sichuan Agricultural University, Chengdu, Sichuan China; 11 U.S. Geological Survey, Northern Rocky Mountain Science Center, Bozeman, MT USA; 12 University of Zurich, Zürich, Switzerland; 13 IREC (Universidad de Castilla – La Mancha & CSIC), Ciudad Real, Spain

**Keywords:** Sarcoptic mange, Wildlife, *Sarcoptes scabiei*, Disease ecology

## Abstract

Sarcoptic mange is a globally distributed disease caused by the burrowing mite *Sarcoptes scabiei*, which also causes scabies in humans. A wide and increasing number of wild mammal species are reported to be susceptible to mange; however, the impacts of the disease in wildlife populations, mechanisms involved in its eco-epidemiological dynamics, and risks to public and ecosystem health are still unclear. Major gaps exist concerning *S. scabiei* host specificity and the mechanisms involved in the different presentations of the disease, which change between individuals and species. Immunological responses to the mite may have a relevant role explaining these different susceptibilities, as these affect the clinical signs, and consequently, the severity of the disease. Recently, some studies have suggested sarcoptic mange as an emerging threat for wildlife, based on several outbreaks with increased severity, geographical expansions, and novel wild hosts affected. Disease ecology experts convened for the “International Meeting on Sarcoptic Mange in Wildlife” on 4–5 June 2018, hosted by the Department of Fish and Wildlife Conservation at Virginia Tech in Blacksburg, Virginia, USA. The meeting had a structure of (i) pre-workshop review; (ii) presentation and discussions; and (iii) identification of priority research questions to understand sarcoptic mange in wildlife. The workgroup concluded that research priorities should be on determining the variation in modes of transmission for *S. scabiei* in wildlife, factors associated with the variation of disease severity among species, and long-terms effects of the mange in wildlife populations. In this note we summarize the main discussions and research gaps identified by the experts.

## Background

Sarcoptic mange is a common, highly contagious skin disease of mammals caused by the burrowing mite *Sarcoptes scabiei* [1]*.* Clinical signs of sarcoptic mange are characterized by hair loss, epidermal crusts, and pruritic dermatitis, and may be present as an acute or chronic process. Impacts in hosts vary widely among mammal species by factors including mite lineage and host immunity [1, 2]. Sarcoptic mange is still being reported in new geographical areas, new animal species, and with new clinical presentations. Empirical evidence demonstrates sarcoptic mange as an emerging wildlife disease in some instances, potentially representing a threat to some wild populations, and an established endemic disease in many other cases [3, 4]. However, the potential long-term effects of mange in wildlife and the causes of the development of epidemic and endemic patterns are still poorly understood [5].

## Overview of the meeting

**Fig. 1:**
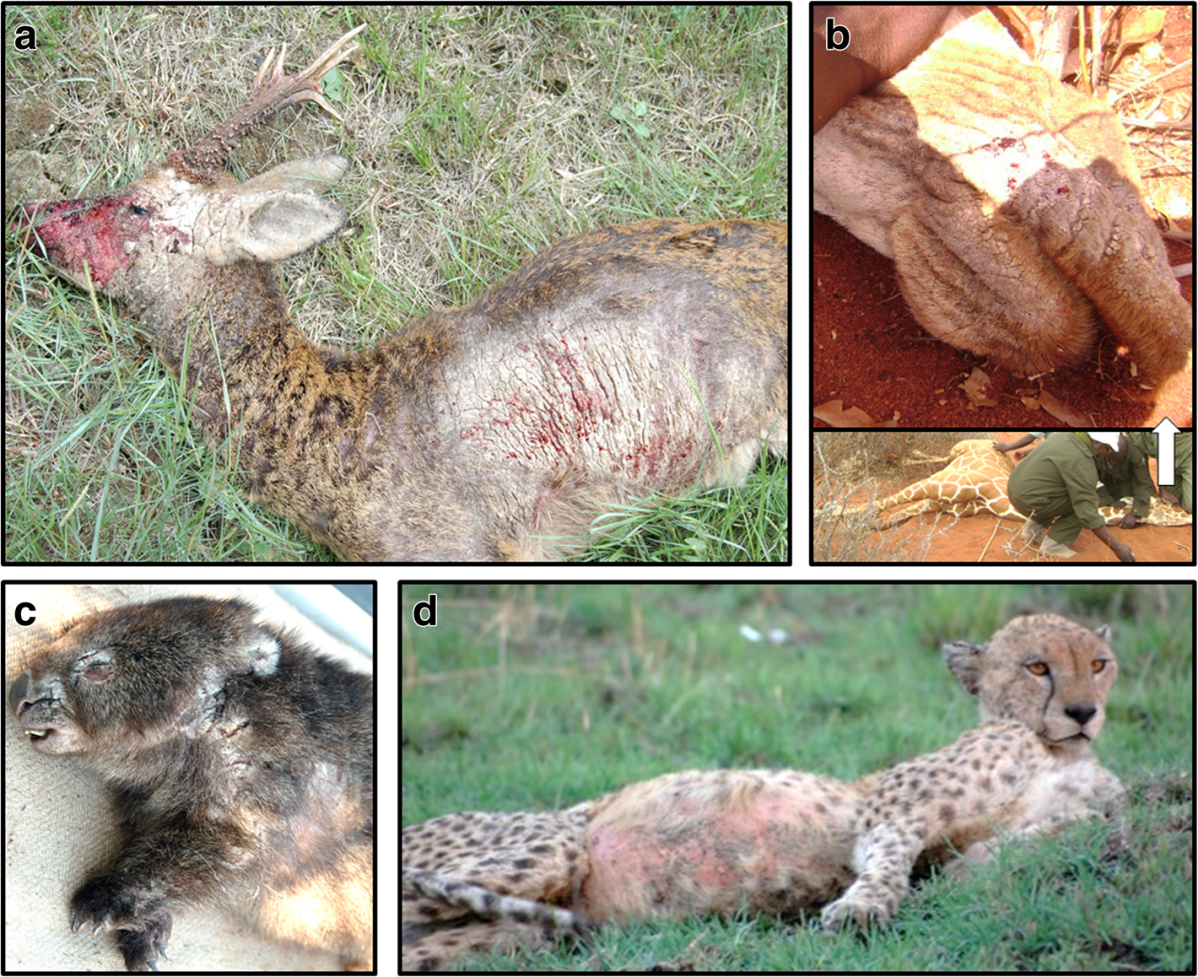
Different wildlife species affected by mange. **a** Roe deer (*Capreolus capreolus*) severely affected by mange (e.g. see hair loss in head, neck, and thorax) in Europe. **b** Giraffe (*Giraffa reticulata*) found affected by mange in Africa (inset: a close up to the animal’s mouth affected by mange). **c** Bare-nosed wombat (*Vombatus ursinus*) infected by mange in Tasmania; note the crusted lesions in the face and shoulder. **d** Cheetah (*Acynonyx jubatus*) found infected with mange in Africa; note the loss of hair in the abdomen

Due to the increasing reports of sarcoptic mange affecting wildlife around the globe (Fig. 1), a group of researchers met during June 4–5, to discuss the current global situation regarding sarcoptic mange in wildlife populations. This report summarizes the main discussions and conclusions raised during the meeting entitled “International Meeting on Sarcoptic Mange in Wildlife”, organized and hosted by the Department of Fish and Wildlife Conservation at the Virginia Polytechnic Institute and State University (Virginia Tech) and supported by funding from the Global Change Center (N° 177219).

**Fig. 2:**
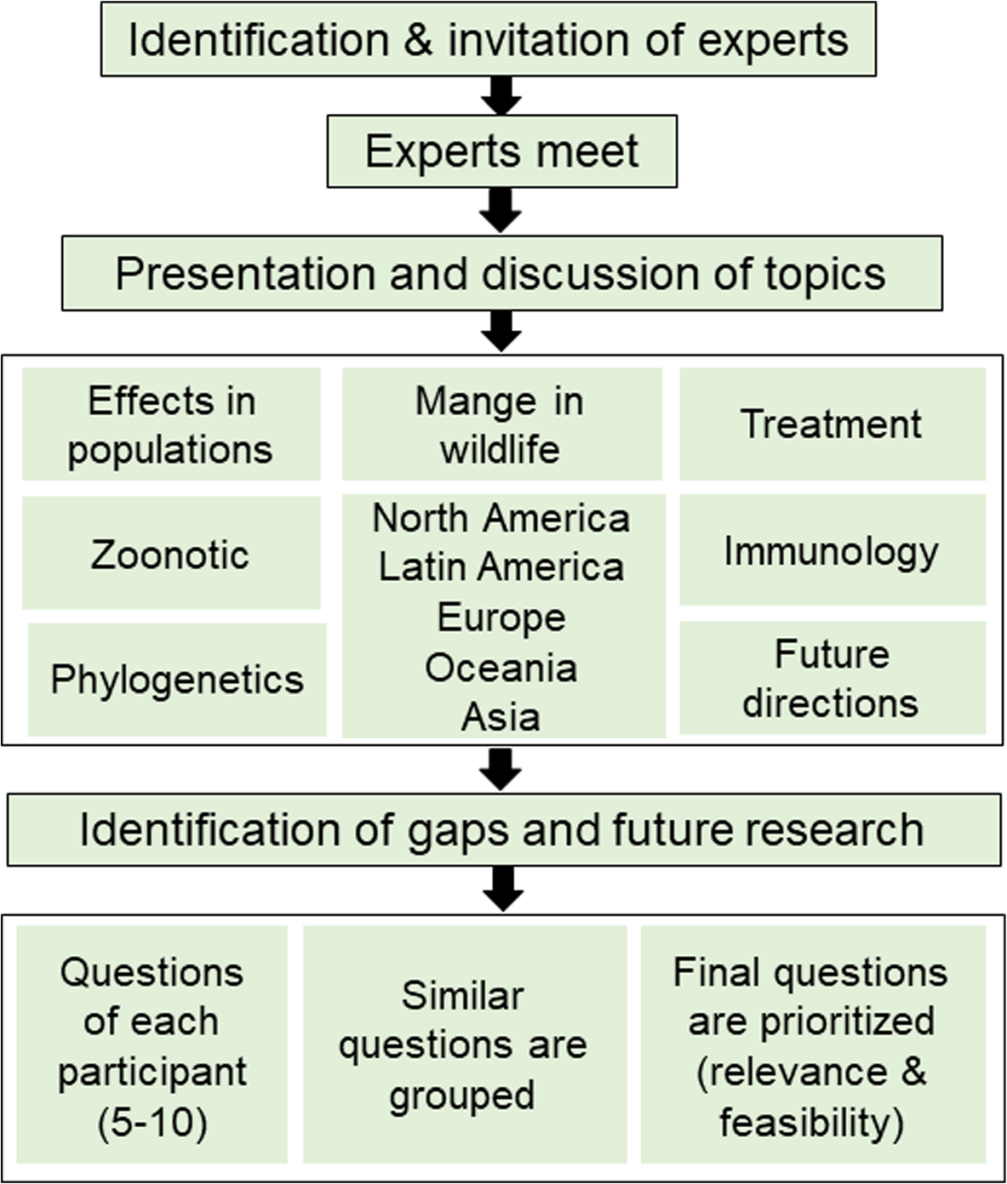
Workshop framework. The first component of the workshop included discussions regarding the state of knowledge of mange in wildlife, followed by a group session to define new research lines

A total of 13 participants presented different aspects of sarcoptic mange, information on state-of-the-art research and outbreaks management. Participants represented academic, federal, state, and non-government institutions from four continents, providing a wide spectrum of approaches to the problem. The main objectives of the meeting were to (i) generate a scoping study of recent advances in the understanding of mange in wildlife, which included discussions regarding new host species, novel geographical areas affected, factors associated with mange outbreaks, and effects of mange in the affected wildlife populations, among others; and (ii) identify major information gaps, which included generation of research questions to be addressed with identification of their priority and feasibility. To accomplish these objectives, the meeting was divided into twelve sessions, preceded by two months of online discussions before the meeting. The first day of the meeting consisted of logistic instructions, plenary discussions, and the development of a scoping study [6] to map the current lines of research in mange. The second day we discussed knowledge gaps and future research topics (Fig. 2). The presentations were sub-structured by geographical region and research area.

## Session 1: Negative effects of parasites on wildlife (Moderator: Dr Paul Cross)

Mange is a contagious disease with characteristic and obvious clinical signs. Sarcoptic mange has negatively affected wildlife globally, specifically grey wolves (*Canis lupus lupus)*, red foxes (*Vulpes vulpes*) and coyotes (*Canis latrans*) in the USA, and red foxes in Europe. Current studies and experimental data show that negative effects may be observable at the population level (e.g. reduction in population densities, removal of juveniles), or at the individual level, such as increased heat loss. These individual impacts may produce behavioral shifts towards decreased movements, increased activities during warmer parts of the day, and increased food consumption, among others. Mange in wildlife species has a general epidemiological pattern, characterized by an initial epizootic phase (with higher prevalence and mortality), followed by an endemic cycle of lower prevalence and potential fade-out. The intensity and impact of the disease appears to vary dramatically among different host species.

### Research gaps identified

- What are the drivers that result in fade out or low endemicity of mange?
- How do immunological, ecological, behavioral and population differences change how mange impact animals across a host species?
- What are the prognostic factors for severe mange infestation at the individual, group and population level?
- What is the relationship between mange infestation and co-infestations/infections with other parasites?

## Session 2: Sarcoptic mange in western North America (Moderator: Dr Emily Almberg)

**Fig. 3:**
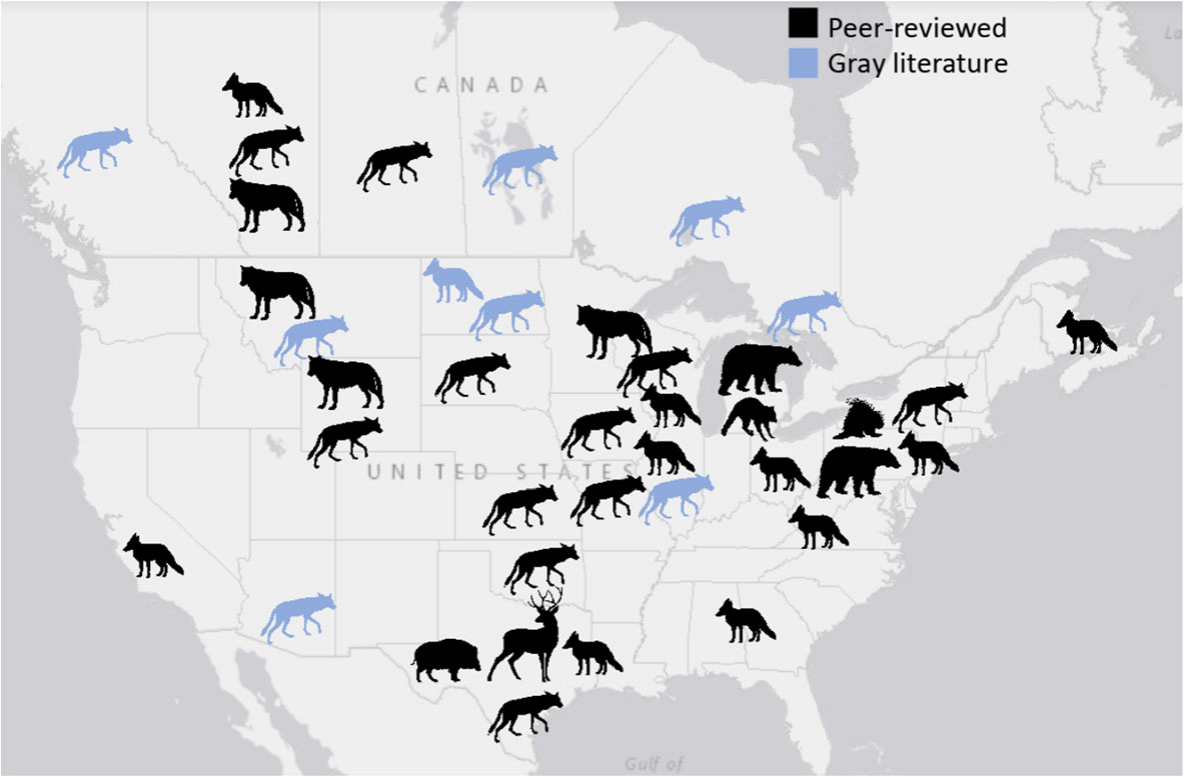
Coarse overview of the areas and species reported infected with mange in the northern North America. The map shows patterns of mange distribution from scientific (black) and gray literature (blue). Silhouettes represent species where mange has been reported

Reports of sarcoptic mange in peer-reviewed and gray literature demonstrate that the mite is widely distributed across North America, with most infections reported in the canid family (i.e. wolves, coyotes and foxes), but also among American black bears (*Ursus americanus*), raccoons (*Procyon lotor*), porcupines (*Erethizon dorsatum*), white-tailed deer (*Odocoileus virginianus*) and wild boar (*Sus scrofa*) (Fig. 3). Studies report both endemic and epidemic conditions, with common observations of outbreak severity peaking during winter and spring months and with high host densities. In North America, there are few reports of mange in far northern latitudes and in extremely arid environments, potentially reflecting a lack of historic disease invasion or low susceptible host densities. Aside from these general observations, little is known regarding the biogeographical patterns of the disease, including paths of spread, mite lineages, and environmental drivers of epidemics. In the case of western North America, geographical barriers such as Rocky Mountains may impose an element to be considered as a limiting feature. However, no research has been conducted to understand the current spatial distribution of mange across the North American continent.

One of the earliest written reports of sarcoptic mange in North America dates to 1909, when the veterinarian for the state of Montana in the United States intentionally infected up to 200 wolves and coyotes with sarcoptic mange and released them to assist with predator control efforts [7]. More recently, sarcoptic mange has been closely studied in the Yellowstone National Park, Wyoming, where *S. scabiei* invaded the reintroduced wolf population in 2007 [8–10]. From these studies, we have found that wolves infected with *S. scabiei* may undergo behavioral changes to cope with the costs of infection, including decreased movement, selection of warmer habitats, increased food consumption, as well as increased social reliance on fellow pack members. Research has also explored the potential predictors of sarcoptic mange severity and associated mortality. Current studies show no associations between individual risk and severity of wolf mange infestations with pack size, age, sex, coat color. Previous infections do not appear to protect individuals from re-infection. Short-term population declines were noted during the initial outbreak of mange, but long-term impacts on population size and resiliency remain an understudied area.

### Research gaps identified

- What drives variation in mange infestation among equally exposed individuals?
- What factors drive the seasonality of mange severity seen in wolf infestations?
- What is the prevalence and impact of mange in areas where there has been no focused reporting or research efforts?
- Does host adaptation affect patterns of mange infestation seen in sympatric host species?
- When should we consider disease management or intervention for mange?
- What are the drivers of mange outbreaks in wildlife?
- Are epidemics driven by new infections in novel/uninfested hosts, or are they the result of changes in host and environmental factors?
- Are there any gaps in mange distribution in wildlife across North America, and if so, what is the reason for non-mange endemic areas?

## Session 3: Sarcoptic mange in eastern North American wildlife (Moderator: Dr Kevin D. Niedringhaus)

Wildlife species in eastern North America, such as the red fox, gray fox (*Urocyon cinereoargenteus*), coyote, white-tailed deer, American black bear, gray squirrel (*Sciurus carolinensis*), fox squirrel (*Sciurus niger*) and the North American porcupine are all affected with mange. Some species are infested specifically with *S. scabiei*, while others are affected by other mites from the genera *Notoedres*, *Demodex*, *Chorioptes*, *Psoroptes* or *Otodectes.* Although major efforts exist in sampling white-tailed deer, there are only a few positive cases in this species, revealing the asymmetrical susceptibility among hosts. Sarcoptic mange is emerging in many species globally, and in North America, mange is becoming more common in the American black bear. Only a few studies have been published in this emerging system. There are new tools to improve diagnoses; however, multiple skin scrapes are still considered the most sensitive test for mite identification and disease diagnosis [11]. Unlike mange in many other species, the highest concentrations of mange in bears are not centered around urban or suburban areas [12]. Lastly, the emergence of mange in bears may not be a result of a new, genetically distinct, or highly virulent mite species based on limited gene targets [13]. Additional research regarding mange in black bears is needed to understand the sudden emergence of the disease in this previously-considered unusual host.

### Research gaps identified

- Is there a role for indirect transmission of mites between bears *via* carcasses or other fomites?
- How can field serology be utilized to determine mange exposure in black bear populations?
- Are bears only transmitting mites between themselves or are other sympatric species involved?
- Why are there fewer reports of sarcoptic mange in cervids, bovids, mustelids and felids in North America compared to other continents?

## Session 4: Mange (*Sarna*) in Latin America (Moderator: Dr Francisca Astorga)

While mange is common in dogs and livestock in Latin America and has been described in at least 24 wildlife species in this region, data on the disease are very scarce, resulting in unknown impacts on wild populations. Since it occurs so often in dogs, it is currently not widely reported, and is not considered a notifiable disease [14]. Camelids are one group with more frequent reports concerning mange, as it causes economic losses to small farmers (e.g. damage of leather, loss of wool, mortality) [15, 16] and may represent an emerging threat to wild native camelids (e.g. *Lama glama guanicoe*) [17]. There is a growing concern about mange transmission between wildlife and livestock, with questions centered on which species is the source of infestation. Mange has also been suggested as the most important disease in the native rodent capybara (*Hydrochaeris hydrochaeris*) in captivity and in free-ranging populations [18]. Additionally, it is noted that most wild canids in Latin America are solitary animals, or tend to live in small families, which is different from North America where canids occur in larger packs. However, a few outbreaks in native canids have been reported [19–21]. The source of these outbreaks is unknown, with potential transmission from domestic dogs or from infected prey [21]. In general, there is a lack of information concerning sarcoptic mange, and grey literature such as theses or institutional reports may be considered as a relevant complementary resource, e.g. [22].

### Research gaps identified

- What is the most effective and feasible way to obtain more data and implement monitoring solutions to determine how mange is impacting wildlife?
- How does disease transmission in canids from North America differ from disease transmission in Latin American canids (social *vs* solitary/small family?)
- Is mange in Latin America primarily originating from wildlife and being transmitted to livestock and other domestic animals, or *vice versa*?
- What role do dogs play in the transmission of mange to other dogs, domestic livestock and wildlife in Latin America?
- Is there an intraguild transmission, a prey-predator transmission, or a combination of the two transmission pathways?

## Session 5: Sarcoptic mange in Europe (Moderator: Dr Christian Gortázar)

Mange in Europe is recorded in several species, and is widespread in European carnivores, wild ruminants, wild boar and lagomorphs. However, mange only has increased relevance in carnivores with regards to red fox and Arctic fox (*Alopex lagopus*). Mange seems to have an endemic cycle in most local hosts, particularly in the Iberian Peninsula, being a part of the natural cycles of the communities. In the long term, wildlife populations in Europe seem to be able to recover and remain stable in number. This pattern suggests that mange control in wildlife in Europe should only be implemented in exceptional situations. A relevant topic is the host-specificity of the mite: in Europe, mange mites seem to affect ungulates much more as compared with North America ungulates (Fig. 1). In North America, ungulates seem to be unaffected or unexposed. Previous studies in Europe have suggested a taxon-specificity (ungulate- and carnivore- cluster) [23]; however, studies have not been conducted in the Americas.

### Research gaps identified

- What are the dynamics involved in the shift from epidemic to endemic cycle of sarcoptic mange in wildlife populations?
- How do factors such as co-infections, health condition, and genetics influence mange infestation in wildlife?
- How can mortality due to mange be properly assessed and differentiated from other causes of death?
- Is sarcoptic mange a threat for biodiversity conservation? Which species or populations should be considered for intervention?

## Session 6: Mange in Oceania (Moderator: Dr Scott Carver)

In Oceania, Australia is the only country in which sarcoptic mange seems to be a real issue in wildlife (there are only two chiropteran native terrestrial mammals in New Zealand, and mange reports were rare to non-existent elsewhere in Oceania). Mange has been reported in many native species in Australia (e.g. koalas, *Phascolarctos cinereus*; dingoes, *Canis lupus dingo*) but is known for having the greatest impacts on wombats [5, 24]. Of the three extant wombat species, the bare-nosed wombat (*Vombatus ursinus*, a.k.a. common wombat) is the most impacted, experiencing widespread endemic disease, with sporadic disease outbreaks and localized population declines [25] (Fig. 1). The southern hairy-nosed wombat (*Lasiorhinus latifrons*) is also impacted [26], but to a lesser extent, and *S. scabiei* may represent a major threat to the northern hairy-nosed wombat (*L. krefftii*), which is critically endangered. In order to assess and develop wombat conservation strategies, recent efforts have focused on individual and population impacts of mange in wombats [25], mange control mechanisms, molecular epidemiology of the mite [27, 28] and a comparison of diagnostic methods [29].

Individual impacts of mange in wombats include aberrant behavior, inefficient thermoregulation and environmental heat loss, and increased metabolism, all of which contribute to changes in wombat foraging and resting patterns [25, 30]. During disease outbreaks, recent research has shown wombat population densities to be inversely related to mange prevalence [25]. There have also been recent experiments evaluating in-field population-scale mange control using topical cydectin treatment of wombats, with the results in preparation for publication. Investigations into the molecular epidemiology of *S. scabiei* have revealed close genetic association between mites derived from wombats, koalas and humans [27]. Recent work has greatly expanded the *S. scabiei* genetic material from Australia and shows multiple circulating haplotypes in mammals across the continent (and globally) implicating multiple *S. scabiei* invasion events to Australia, originating with European settlers to the continent [31].

### Research gaps identified

- What are the best treatment options and delivery strategies that would result in control, and ultimately, significant reduction of mange in wombats?
- How do the environmental conditions affect mange transmission?
- What factors shape the range of animal hosts affected by the *S. scabiei* mite?
- Are there differences in immune gene expression between healthy wombats and those infested with mange, and if so, how is immune gene expression associated with severity of disease within skin and tissues?

## Session 7: Sarcoptic mange in Asia (Moderator: Dr Yue Xie)

Sarcoptic mange has been reported throughout Asia in both wildlife and captive species. While cases have been reported in Japan, China, Korea and Israel, the number of wildlife cases has been deemed limited, with outbreaks occurring in specific geographical locations. Ruminants are noted as being the most prominent wildlife species infested with *S. scabiei*. Infested ruminants include mountain gazelles (*Gazella gazella*), Nubian ibex (*Capra nubiana*), barbary sheep (*Ammotragus lervia*), elands (*Taurotragus oryx*), Arabian oryx (*Oryx leucoryx*), Alpine ibex (*Capra ibex*), blue sheep (*Pseudois nayaur*), tufted deer (*Elaphodus cephalophus*), gorals (*Naemorhedus goral*) and serows (*Capricornsis sumatraensis*). Outbreaks of sarcoptic mange in raccoon dogs (*Nyctereutes procyonoides*) have been reported since 1993. Mange infestation in captive wildlife species such as red panda (*Ailurus fulgens*), sika deer (*Cervus nippon*), Chinese mountain cats (*Felis bieti*) and rhesus monkeys (*Macaca mulatta*) have also been reported in China.

### Research gaps identified

- Why are cases of mange in wildlife in Asia deemed to be few in number compared to other continents?
- Is the limited number of outbreaks due to limited reporting or surveillance data for wildlife?
- Are there any endangered species currently impacted that require intervention efforts?
- What is the relationship between mange in domestic species and mange cases in wildlife and/or captive wildlife species?

## Session 8: *Sarcoptes scabiei* in humans: a public health perspective (Moderator: Kimberly Wingfield)

Domestic animals, wildlife, and humans can all be infested with the *S. scabiei* mite, which is termed mange in animals or scabies in humans. Human scabies occurs when individuals are infested with the human mite (*S. scabiei* var. *hominis*), and zoonotic scabies occurs when infested with an animal host-specific variant of the mite [32]. Human scabies is more readily transmissible between humans and is generally more clinically severe for humans as compared to zoonotic scabies [33–35]. Thus, treatment is required for human scabies, whereas zoonotic scabies is generally transient and self-limiting [35, 36]. In all cases of scabies, risk of secondary infection is increased due to disruption of the skin’s protective layer [36]. For both human scabies and zoonotic scabies, clearing of the mite from the environment is key in preventing further transmission of the disease [35, 37]. Those with occupational hazards for *S. scabiei* mite infestation (i.e. human health care workers or animal care professionals) should take extra precautions such as donning protective clothing to prevent mite infestation. Additionally, in cases of zoonotic scabies, treatment of the animal for mange is a necessary step to prevent the continued spread of mites to humans and to other animals.

### Research gaps identified

- How can proper diagnosis and determination of mite origin be improved in order to differentiate scabies from other dermatological diseases, and/or differentiate classic human scabies from zoonotic scabies?
- Is it possible for a human with zoonotic scabies to further transmit the mite(s) to other humans or back to another animal?
- What explains the variability in disease severity among people infected with *S. scabiei*?
- What methodologies can be developed and implemented in order to increase monitoring and reporting of zoonotic scabies?
- What percentage of scabies cases are zoonotic in origin?
- Which *S. scabiei* lineages infest humans more frequently?

## Session 9: Treatment of mange in wildlife (Moderator: Dr Peach Van Wick)

Treatment for mange in wildlife is similar to that for domestic animal species (e.g. routes and options of treatment); however, the feasibility of treating wildlife poses certain challenges. For example, a common treatment option for mange in wildlife is the administration of two doses of ivermectin, approximately two weeks apart. Although effective, this option necessitates the animal(s) be held in a captive environment or re-captured in the field to receive the full treatment. Another consideration in managing mange in wildlife is deciding if treatment should be conducted at the individual or population level or if treatment should be pursued at all. Additionally, the available treatment options for mange (i.e. avermectins, milbemycins and isoxazolines) were developed for the use in domestic animals or humans, not for wildlife. Thus, treatment options and protocols are often extrapolated from domestic species assuming effectiveness in wildlife. Additional concerns such as drug residues in edible tissues must be considered if the wildlife is hunted for human consumption.

### Research gaps identified

- What is the feasibility of treating wildlife, and is it necessary?
- Should treatment of mange be targeted only for isolated or endangered populations, or should it be targeted for those populations with highest infestation risk that could potentially spread mange to other species?
- Should treatment of secondary issues of mange (e.g. bacterial skin infections, emaciation) also be of treatment focus in wildlife?
- Is vaccination an appropriate and more proactive treatment option?
- What procedures can be implemented to ensure decreased consumption risk of mange treatment residues in wildlife that are considered as game?
- What considerations should be developed and implemented to address environmental contamination from mange treatment in wildlife?

## Session 10: Immunology of mange in wildlife (Moderator: Dr Giovane R. Sousa)

The immune response to *S*. *scabiei* in wildlife is poorly understood. However, a consistent pattern is that an initial mange infection leads to suppression of the immune system possibly through mite secreted substances [38]. These mite antigens might cause an anti-inflammatory immune response and this inhibitory mechanism seems to help mites to survive in the host skin and establish a population during the early stages of the disease. Then, there is a shift to allergic and inflammatory responses and symptoms of the disease are manifested [39]. Previous studies have shown that the response to mange differs among distinct host species. For instance, dogs, humans and rabbits acquire full immunity. Conversely, red foxes do not seem to have immune response to mange due to lack of memory T-cells after initial infection. Additionally, the immune response can be uneven within the same species due to the sex of the animal, or previous exposure status. Mange in American black bears has become an emerging concern and thus the hypothesis that mite-mediated immune response is associated with the pathogenesis of mange in free-ranging black bears has been investigated. In preliminary data, inflammatory genes NF-kB, IFN-γ and TNF-α appear to be upregulated in mange-infested black bears compared to healthy black bears not infested with mange. Interestingly, a wide variation could be observed within the *S*. *scabiei*-infested group for its expression of NF-kB and IFN-γ, important genes for activation of the inflammatory response. It is possible that those animals with increased activation of these genes may well be those who presented a more severe progression toward disease involvement. With regards to genes IL-10 and IL-4, which regulates the expression of those anti-inflammatory cytokines, no significant difference in expression between healthy black bears and those infested with mange have been observed. Taken together, these results suggest that parasite-mediated immune response in free-ranging black bears is characterized by a predominantly inflammatory immune response, which might associate with mange severity.

### Research gaps identified

- How can the effect of immunological responses on mange severity be studied in wildlife?
- What host-parasite interaction factors are the primary drivers for both positive and negative clinical outcomes of mange in wildlife?

## Session 11: Phylogenetics of wildlife mange (Moderators: Drs Samer Angelone & Luca Rossi)

Sarcoptic mange is a very old disease, first described for humans in the 11th century. There are two important events in the history of scabies infestation discovery in humans: in the early 11th century it was proposed that the clinical symptoms of mange were caused by a parasite [40]; and in the 17th century occurred the identification of *Sarcoptes* by Francesco Redi Arezzo [41]. A more recent discovery is host-taxon derived *Sarcoptes*, as grouped by herbivore, carnivore and omnivore hosts [42]. Transmission mechanisms between animal hosts, and the role that fomites play have been studied, but more research to elucidate these mechanisms is needed [43]. Several studies have been developed recently concerning the epidemiology and genetics of sarcoptic mange in wildlife [44]. In Europe, a taxon-associated cluster was found, i.e. transmission seems to be mainly among species from the same taxonomic group (herbivores *vs* carnivores) [42]. In contrast, in the Masai Mara Reserve (Africa), transmission seems to be occurring between prey and predators (Fig. 1) [45]. In the meantime, in order to obtain more data, scientific communities should consider Citizen Science, to invite tourists and the local community to report potential cases of mange in wildlife [46]. Additionally, data integration groups such as those of the Sarcoptes-World Molecular Network, may help to provide more information on *S. scabiei* transmission *via* information exchange efforts and building of databases for research analyses [47].

### Research gaps identified

- How can phylogenetic data be used understand sarcoptic mange transmission?
- How does *S. scabiei* move between and among different host species?
- How can problems in research such as unstandardized epidemiological methodology, small sample sizes of research papers on the topic, and lack of outgroups be overcome when studying mange?
- How effective are mange detector dogs and PCR in diagnosis mange in wildlife? Are these solutions feasible?

## Session 12: Research questions and future directions (Moderator: Dr Luis E. Escobar)

The final session included a participatory determination of mange in wildlife research needs. During the second day, participants independently wrote up to ten key questions, which all participants then grouped into clusters based on similarities. These clusters were then summarized, organized, and discussed in order to classify the final list of questions, with sub-components, in terms of their relevance to the understanding of mange in wildlife (Table 1).

**Table 1:** Main questions (and sub-elements) concerning wildlife mange

Question	
(i) What are the transmission dynamics of *S. scabiei* in wildlife?	
Temporal dynamics (epidemic, endemic, contemporary and historical)	
Significance for species conservation	
Pandemic or globally endemic	
Species dynamics (single or multi-species)	
Wildlife-domestic animals-human interface	
Ecosystem level consequences	
(ii) What is the biogeographical history of *S. scabiei*?	
Native range / origins	
Pathways of spread: unintentional (e.g. trade, colonization) and intentional (e.g. host population control)	
Ecological (e.g. host distribution), climatic (e.g. temperature, humidity) and geographic barriers (e.g. oceans, mountains)	
(iii) What is the variation in modes of transmission for *S. scabiei* in wildlife?	
Intra-specific interactions (spatial and temporal variation)	
Inter-specific interactions (trophic, sympatry)	
Environmental effects	
Mite lineages / strains	
(iv) What is the feasibility and anticipated effectiveness of intervention strategies in wildlife?	
Methodological (individual level, population level, environmental)	
Economic	
Indirect impacts	
(v) When should intervention strategies for mange be considered in wildlife?	
Conservation concerns	
Ethical/welfare concerns	
Public health and domestic animal (agricultural and companion) concerns	
Ecological role of the parasite in nature (e.g. natural selection)	
(vi) What shapes the variation of disease severity at intra-specific and inter-specific levels?	
The role of co-infections	
Variation in immune response (e.g. immunological pathways influencing disease progression or control, ordinary *vs* crusted mange)	
Environmental factors	

## Conclusions

Wildlife diseases play a major role in biodiversity conservation [48, 49]. However, effects of most diseases in wildlife species remain poorly understood. Sarcoptic mange is a worldwide distributed disease, which affects mammal species, populations and individuals. Although it is an old disease, there remains much to be learned of its ecology and epidemiology in wildlife. To develop a comprehensive global assessment of mange and the threats it poses to wildlife, further efforts are recommended to concentrate in questions that addresses the major gaps identified here (Table 1). We propose six major research questions that, if explored, will augment our understanding of the natural history of mange in wildlife. Finally, mange is an ideal disease to implement One Health research because it can affect domestic and wildlife animals and humans and the habitat can play a role in the transmission. Mange is also an excellent system to better understand host-parasite interactions and the biogeography of ectoparasites.

## Abbreviations

NF-kB: Nuclear factor kappa-light-chain-enhancer of activated B cells
IFN-γ: Interferon-gamma
TNF-α: Tumor necrosis factor-alpha
IL: Interleukin
PCR: Polymerase chain reaction
